# AAV‐mediated gene therapy improving mitochondrial function provides benefit in age‐related macular degeneration models

**DOI:** 10.1002/ctm2.952

**Published:** 2022-08-21

**Authors:** Sophia Millington‐Ward, Naomi Chadderton, Laura K. Finnegan, Iris J.M. Post, Matthew Carrigan, Tom Gardiner, Elisa Peixoto, Daniel Maloney, Marian M. Humphries, Alan Stitt, Thierry Léveillard, Pete Humphries, Paul F. Kenna, Arpad Palfi, G. Jane Farrar

**Affiliations:** ^1^ Smurfit Institute of Genetics School of Genetics and Microbiology Trinity College Dublin Dublin 2 Ireland; ^2^ Queen's University Belfast Centre for Experimental Medicine School of Medicine Dentistry and Biomedical Science Belfast UK; ^3^ Department of Genetics Sorbonne Université INSERM CNRS Institut de la Vision Paris France; ^4^ Research Foundation Royal Victoria Eye and Ear Hospital Dublin 2 Ireland

Dear Editor,

With an estimated 196 million people suffering from age‐related macular degeneration (AMD) in 2020 and predicted to increase to 288 million by 2040,^1^ dry AMD, representing 70%–90% of AMD cases, represents an enormous clinical need with no current therapies. We have demonstrated that NDI1 and an optimised version of NDI1 (ophNdi1), a mitochondrial complex 1 equivalent from *Saccharomyces cerevisiae*, provide functional and histological benefit in two murine models of dry AMD as well as benefit in two cellular models of dry AMD. There are no drugs on the market for dry AMD. However, there are currently a small number of candidate gene therapies in clinical trial (clinicaltrials.gov). To our knowledge, this is the first demonstration that a gene therapy directly targeting mitochondrial dysfunction provides functional benefit in in vivo models of dry AMD, making this a novel approach to treating this devastating condition.

Dry AMD is characterised by the formation of drusen between Bruch's membrane (BM) and the basal lamina of the retinal pigment epithelium (RPE) and atrophic changes in the choriocapillaris followed by the death of photoreceptors in the macula and geographic atrophy, with a related loss of central vision. AMD is multifactorial with genetic and environmental factors known to contribute to the disease.^2^ Although underlying mechanisms involved in AMD are not fully understood, mitochondrial dysfunction leading to increased oxidative stress in the RPE, DNA damage and impaired mitophagy are known to contribute to RPE and photoreceptor cell death.^3^ Both the RPE and photoreceptors have been shown to display mitochondrial complex 1 (of the electron transport chain) deficiency.^4^

The *Cfh^−/−^* mouse^5^ has been widely used as a dry AMD model and aged *Cfh^−/−^* mice have been reported to display impaired visual function, thinning of the retinal outer nuclear layer, changes in BM and basal laminar deposits (BlamDs).^5^, ^6^In this study, we also observed electroretinography (ERG) deficits in aged *Cfh^−/−^* mice (Figure 1A–D, Table S1), but no changes in BM or BlamDs were apparent. However, cone photoreceptors exhibited disorganised outer segments, and substantial mitochondrial alterations compared to cones of control mice. Cone mitochondria appeared shrunken and fragmented and the cytoplasm of inner segments swollen and electron‐lucent. These changes in cone histology have not previously been reported and indicate mitochondrial dysfunction (Figure 1E–J).

**FIGURE 1:**
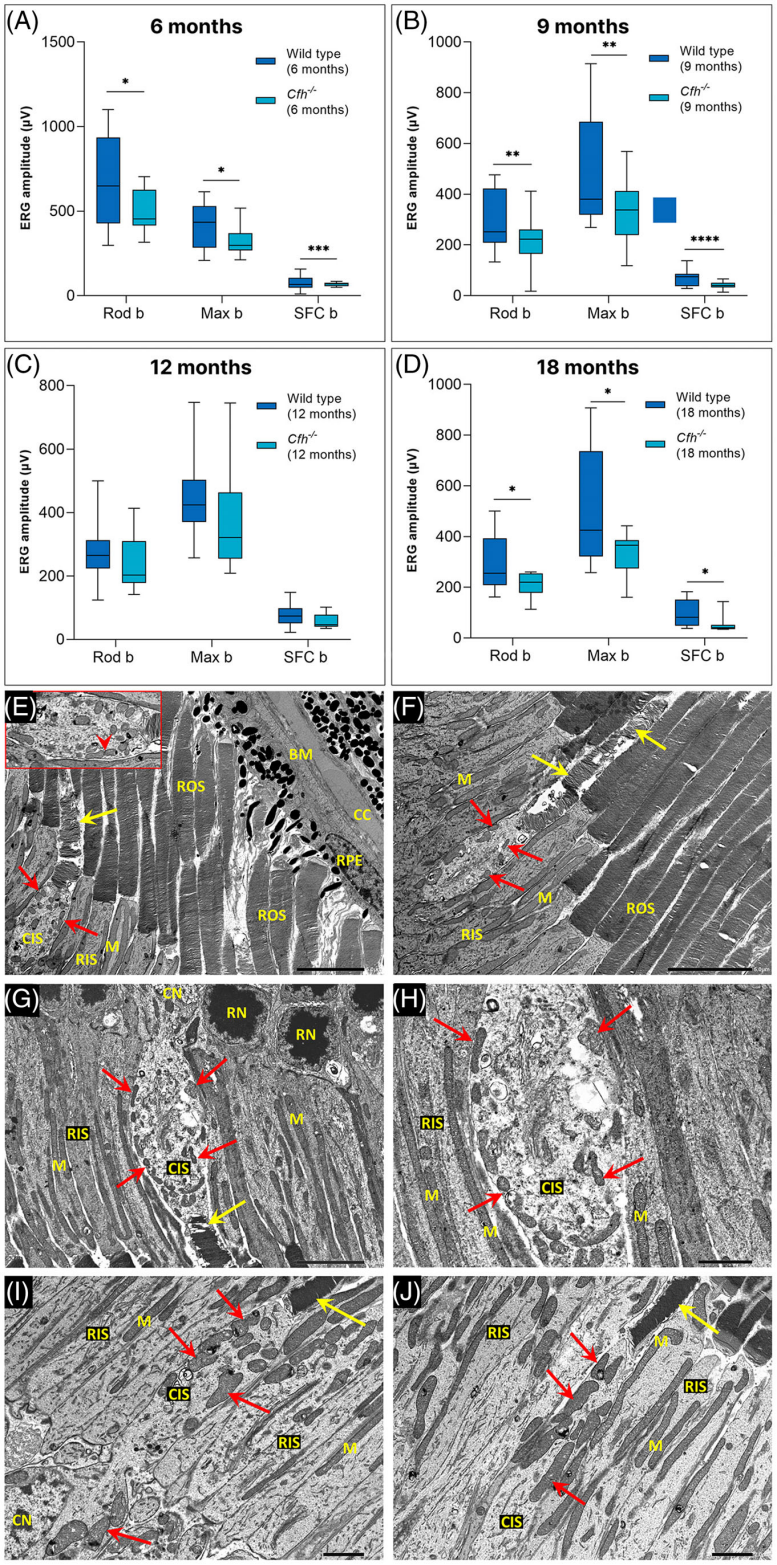
*Cfh^−/−^* murine model. (A–D) Electroretinography (ERG) analysis on C57BL/6J and *Cfh^−/−^* at 6, 9, 12 and 18 months of age. At all ages, (A) 6 months, (B) 9 months, (C) 12 months and (D) 18 months, *Cfh^−/−^* mice displayed significantly reduced Rod b, Max b and single flash cone (SFC) b responses compared to C57BL/6J mice. ^*^ *p* < .05, ^**^ *p* < .001 and ^***^ *p* < .0001; 2‐sample *t*‐test. (E–J) Transmission electron microscopy (TEM) on 12 months C57BL/6J and *Cfh^−/−^* mouse retinas. (E) The retinal pigment epithelium (RPE) in *Cfh^−/−^* mice appeared normal with intact basal infoldings of the basolateral plasma membrane and no evidence of electron‐dense deposits. Bruch's membrane (BM) was of comparable thickness to controls wild‐type (WT) C57BL/6J controls (I and J) and showed no electron‐dense deposits. The choriocapillaris (CC) was intact, and the endothelium was densely fenestrated at the RPE interface. The cone cells in *Cfh^−/−^* mice (E–H) exhibited the disorganization of the outer segment (OS) membranes (yellow arrows in E–G). Also, the mitochondria (M) in the cone inner segments (CIS) of *Cfh^−/−^* retinas were irregular in size and shape (red arrows) and generally much smaller than those in adjacent rod inner segments (RIS). Some cone mitochondria appeared to be undergoing fission (E‐inset, red arrowhead). The cytoplasm of the CIS was generally more electron‐lucent than that of rods (E–H) and some appeared swollen (G and H). Cone cells in WT control mice (I and J) showed normal OSs (yellow arrows) and large mitochondria, which in contrast to those in *Cfh^−/−^* mice tended to have a greater diameter (red arrows) than the mitochondria in adjacent rods. CN, cone nucleus; RN rod nucleus

We have investigated the utility of the nuclear‐encoded NDI1^7^ gene as a candidate therapy for dry AMD. NDI1 provided benefit in models of Parkinson's disease, Leber hereditary optic neuropathy and multiple sclerosis.^8^ NDI1 has also been shown to reduce reactive oxygen species (ROS) and oxidative stress in disease models.^7^, ^8^We utilised a codon‐optimised version of NDI1, ophNdi1, which we observed to express ∼3‐fold higher than wild‐type NDI1 in murine retina from recombinant adeno‐associated viral (AAV) vectors following subretinal delivery (Figure S1).

A range of AAV2/8 and AAV2/5 viral doses (1.0 × 10^7^–7.5 × 10^9^ vg) were used to deliver ophNdi1 and NDI1 subretinally to *Cfh^−/−^* mice. Significant and robust functional benefit was observed in 60 aged mice using ERG readouts, as well as reduced ROS, increased nicotinamide adenine dinucleotide (NADH) oxidation and increased cone photoreceptor numbers in treated versus control eyes (Figures 2A–O, S2, S3). Notably, with none of the doses used were negative effects observed even up to 7–9 months post‐injection. In acknowledgement that no model recapitulates all aspects of dry AMD, a second murine model, the well‐established sodium iodate‐induced (NaIO_3_) model,^9^was also treated subretinally with AAV2/8‐ophNdi1 and AAV2/5‐ophNdi1. NaIO_3_, a strong oxidising agent, causes catastrophic damage to the RPE leading to subsequent photoreceptor loss and reduced photoreceptor cell function, including reduced ERG amplitudes when delivered systemically.^10^ Similar to our findings in the *Cfh^−/−^* mouse, subretinally delivered AAV‐ophNdi1 provided robust ERG benefit, as well as improved optokinetic responses and increased cone photoreceptor cell numbers in treated versus control eyes (Figure 2K–O).

**FIGURE 2:**
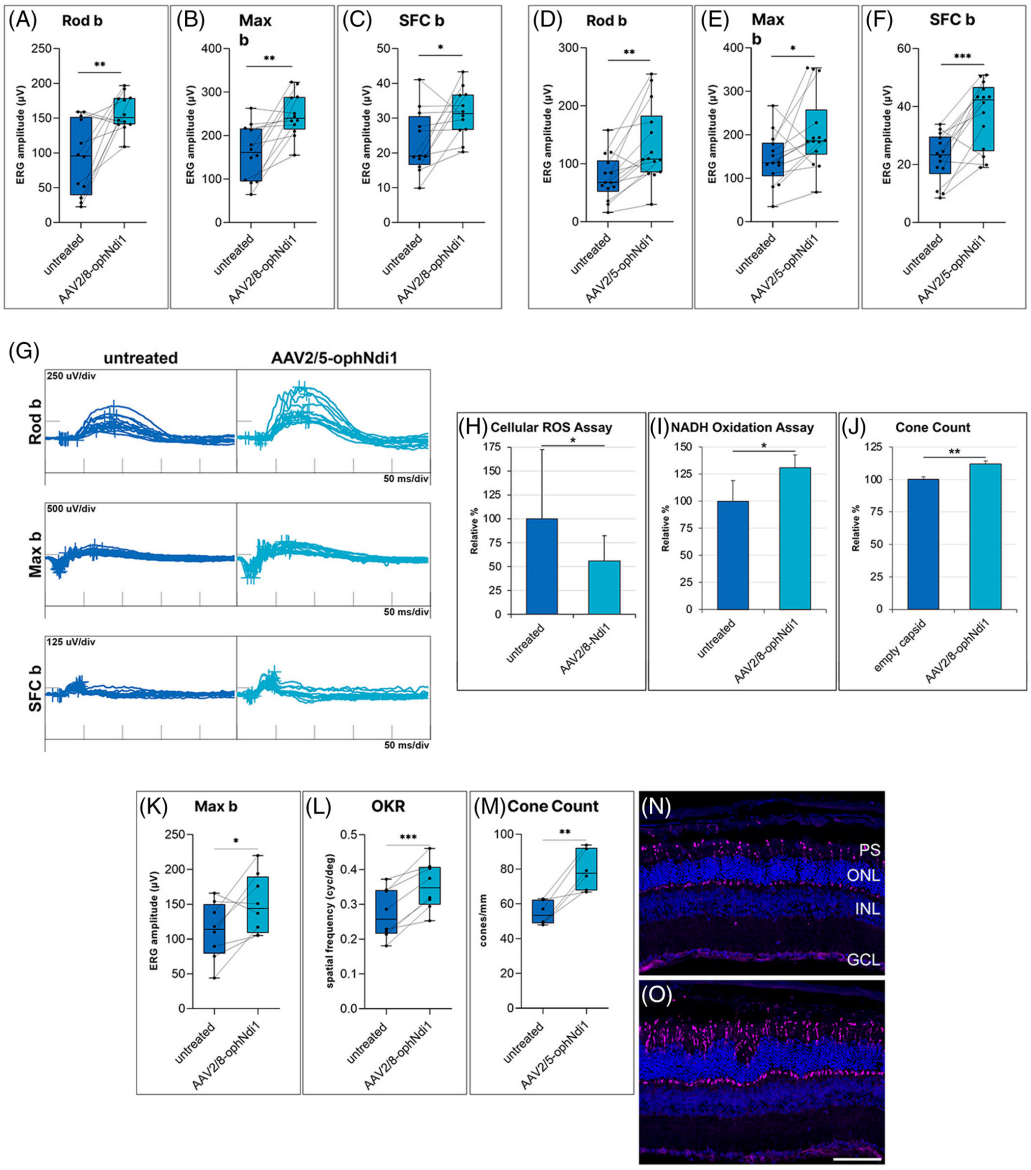
Rescue of the *Cfh^−/−^* genetic and NaIO_3_‐induced murine models. (A–C) Two‐month *Cfh^−/−^* mice were subretinally injected with 1.0 × 10^7^‐vg adeno‐associated viral (AAV)2/8‐ophNdi1 in one eye, whereas contralateral eyes were not uninjected (*n* = 12). Electroretinographies (ERGs) of treated eyes compared to untreated contralateral eyes at 9 months showed significantly greater Rod b (A, 158.1 ± 26.22 vs. 93.24 ± 53.37 μV), Max b (B, 247.7 ± 49.71 vs. 159.7 ± 63.09 μV) and single flash cone (SFC) b (C, 31.43 ± 6.968 vs. 23.03 ± 9.006 μV) responses. ^*^ *p* < .05; ^**^ *p* < .01; paired *t*‐test. (D–G) Two‐month *Cfh^−/−^* mice were subretinally injected with 5.7 × 10^8^‐vg AAV2/5‐ophNdi1 in one eye, whereas the contralateral remained uninjected (*n* = 14). ERGs (D) of treated eyes compared to untreated contralateral eyes at 9 months showed significantly greater Rod‐b (E, 76.0 ± 38.7 vs. 133.4 ± 66.2 μV), Max b (F, 143.8 ± 58.8 vs. 207.1 ± 86.9 μV) and SFC b (G, 22.3 ± 8.27 vs. 37.54 ± 11.44 μV) responses. ^*^ *p* < .05; ^**^ *p* < .01; ^***^ *p* < .001; paired *t*‐test. (H) A CellRox (ROS) assay was performed on retinal cells from *Cfh^−/−^* untreated mouse retinas (*n* = 17) or mouse retinas treated with AAV2/8‐Ndi1 (*n* = 10). Mice were subretinally injected with 2.4 × 10^9^‐vg AAV2/8‐Ndi1 at 2 months of age. CellRox was measured in dissociated retinal cells 3 months post‐injection. Levels in untreated cells were taken to be 100%. CellRox levels in untreated versus treated cells were 100% ± 72.5% and 55.9% ± 26.6%, demonstrating a significant reduction in ROS in AAV2/8‐Ndi1‐treated retinas of 44.1% (2‐sample *t*‐test). (I) NADH oxidation activities in retinas of 14‐month *Cfh^−/−^* mice subretinally injected in one eye with 7.5 × 10^7^‐vg AAV2/8‐ophNdi1 and in the contralateral eye with 7.5 × 10^7^ empty capsids were compared (*n* = 4 retinas per group); levels in untreated retinal samples were taken to be 100%. NADH oxidation activity was 31.0% higher in eyes that received AAV2/8‐ophNdi1 compared to control eyes (paired *t*‐test). (J) Sections of retinas from 1‐year old *Cfh^−/−^* mice that received 1.0 × 10^7^‐vg AAV2/8‐ophNdi1 in one eye and 1.0 × 10^7^ empty vector in the fellow eye were cryosectioned and cones stained with Arr3 immunohistochemistry. Cone numbers in untreated retinas were considered to be 100%. (K and L) Adult 129 S2/SvHsd mice (*n* = 8) were subretinally injected with 7.5 × 10^7^‐vg AAV2/8‐ophNdi1 and 1.0 × 10^8^‐vg AAV2/2‐CAG‐EGFP in one eye. Fellow eyes received an equal volume of PBS containing 1.0 × 10^8^‐vg AAV2/2‐CAG‐EGFP. Three months post‐injection 22‐mg/kg NaIO_3_ in .9% NaCl_2_ was administered via tail vein. (K) One‐week post‐insult, ERG analysis was undertaken; AAV2/8‐ophNdi1‐treated eyes had significantly greater Max b responses than untreated eyes, 150.8 ± 42.6 versus 111.9 ± 41.2 μV. (L) Optokinetic (OKR) analysis 4‐week post‐insult showed better responses in treated versus untreated eyes, .354 ± .0652 cyc/deg versus .273 ± .0667 cyc/deg, respectively. (M) Adult 129 S2/SvHsd mice (*n* = 6) were subretinally injected with 5.7 × 10^8^‐vg AAV2/5‐ophNdi1 and 1.0 × 10^8^‐vg AAV2/2‐CAG‐EGFP in one eye. Control eyes received equivalent volumes of PBS containing 1.0 × 10^8^‐vg AAV2/2‐CAG‐EGFP. Three months post‐injection 50‐mg/kg NaIO_3_ in .9% NaCl_2_ was administered via tail vein. Seven‐day post‐insult mice were sacrificed, eyes fixed, retinas cryosectioned and stained with Arr3 (cone antibody, magenta) and dapi (nuclear stain, dark blue). Sections from untreated (N) and treated (O) eyes had cone numbers of 54.84 ± 6.794 versus 79.26 ± 11.49 cones/mm, representing a 44.5% increase with treatment. ^*^ *p* < .05; ^**^ *p* < .01; ^***^ *p* < .001; paired *t*‐test. Scale bar (O): 100 μm

To interrogate the mechanism behind the observed functional and histological benefit in the treated murine NaIO_3_ model, cellular models utilising NaIO_3_ were investigated; primary porcine RPE (pRPE) cells and ARPE19 cells, a well‐established cell line with some characteristics of RPE. pRPE cells were transduced with AAV2/8‐ophNdi1 and insulted with NaIO_3_. Immunocytochemistry for 8‐OHdG (oxidative stress marker), CPN60 (mitochondrial marker) and phalloidin (selective for F‐actin) showed high levels of oxidative and mitochondrial stress and the absence of actin filaments in NaIO_3_‐insulted versus control cells, indicating severe stress and reduced viability. In contrast, insulted cells transduced with AAV2/8‐ophNdi1 appeared similar to control cells (Figures 3A–O, S4). Similar rescue from NaIO_3_ insult was also observed in ARPE19 cells transduced with AAV2/8‐ophNdi1 (Figures 4A–O, S5). These data suggest that AAV2/8‐ophNdi1 treatment provides significant protection against mitochondrial stress, oxidative damage to DNA and cell death in the cellular NaIO_3_ models.

Additionally, mitochondrial stress tests were performed on pRPE cells transduced with AAV2/2‐ophNdi1 and insulted with NaIO_3_. NaIO_3_ insult significantly reduced basal oxygen consumption rates (OCRs), maximal OCRs and ATP production in cells. However, treatment with AAV2/2‐ophNdi1 significantly increased each of these parameters indicating a rescue of mitochondrial function (OXPHOS, Figure 3P–R). Spare respiratory capacity, the difference between maximal OCR and basal OCR, was reduced with AAV2/2‐ophNdi1 treatment as basal OCR was increased by more than the maximal OCR (Figure 3Q). When pRPE cells were exposed to the complex 1 inhibitor rotenone, OCRs were reduced to background levels in control and NaIO_3_‐insulted cells. However, the addition of rotenone to AAV2/2‐ophNdi1‐treated cells – NDI1 is insensitive to rotenone – had minimal effect on OCR levels, which were substantially maintained (Figure 3S). Notably, similar benefits in bioenergetic profiles were also observed in NaIO_3_‐insulted ARPE19 cells transduced with AAV2/2‐ophNdi1 (Figure 4P,Q, Table S2).

**FIGURE 3:**
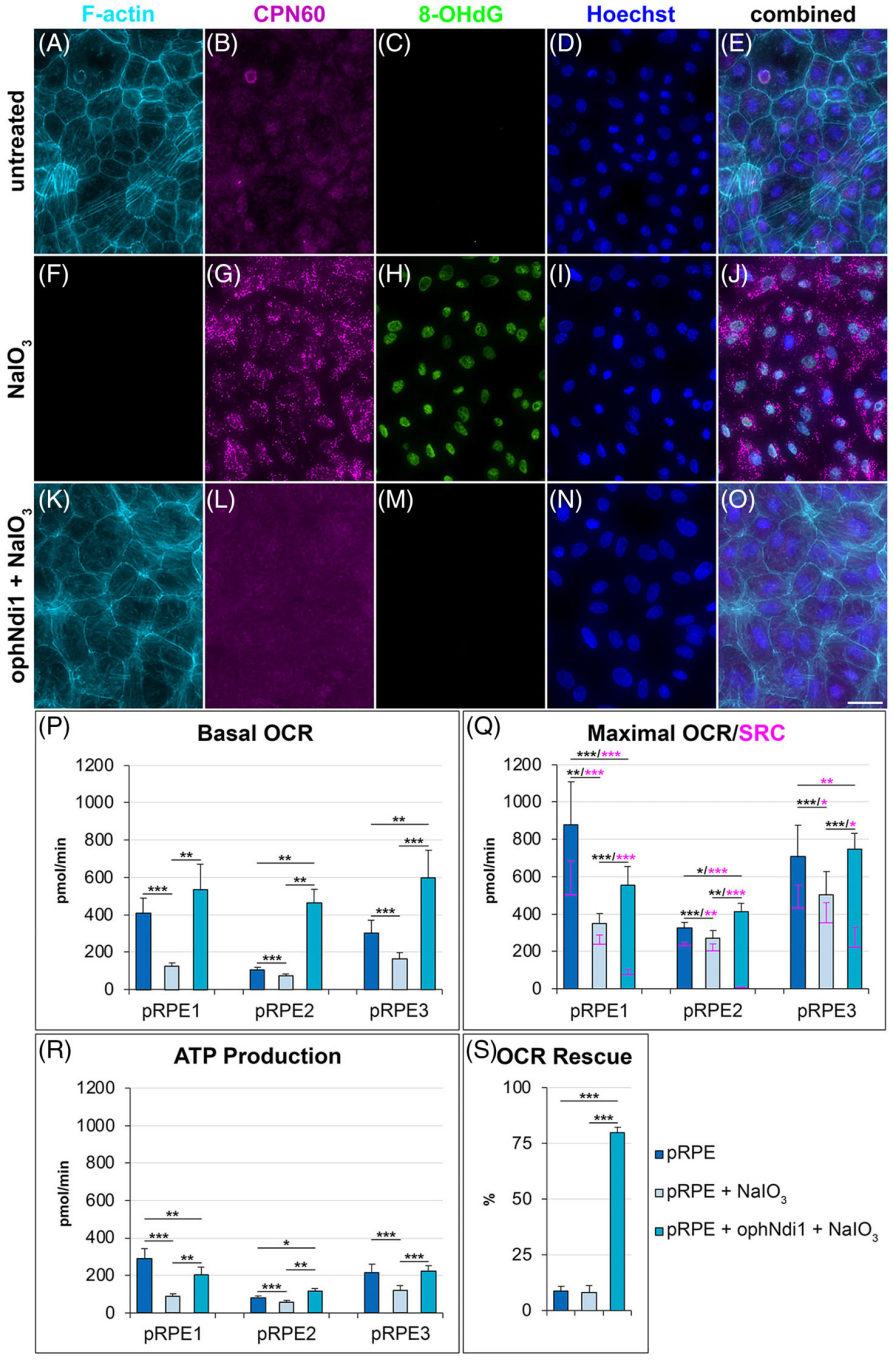
Rescues of primary porcine retinal pigment epithelium (pRPE) cells insulted with NaIO_3_. 7.5 × 10^4^ pRPE cells (from *n* = 3 pigs) were transduced with AAV2/8‐ophNdi1 5‐h post‐seeding (MOI = 5.4 × 10^5^; K–O). Twenty‐eight‐hour post‐transduction cells were insulted with 5‐mM NaIO_3_ (F–O) and 24‐h post‐insult cells were fixed and stained with Phalloidin‐iFluor 647 (F‐actin, light blue), and CPN60 (mitochondrial marker, magenta) and 8‐OHdG‐Alexa Fluor 488 (oxidative stress marker, green) immunocytochemistries; nuclei were counterstained with Hoechst (nuclear stain, dark blue). AAV2/8‐ophNdi‐treated and NaIO_3_‐insulted cells (K–O) were compared to untreated (A–E) and untreated and NaIO_3_‐insulted cells (F–J). Expression of ophNdi1 provides clear rescue and insulted cells treated with the virus have a similar phenotype to untreated control cells. Scale bar (O): 25 μm. (P–S) 5.0 × 10^4^ primary pRPE cells (*n* = 3 pigs; pRPE1–3) were seeded into XFe96 Seahorse plates. The following day a minimum of five wells were transduced with AAV2/2‐ophNdi1 (MOI = 3.4 × 10^5^). Twenty‐eight‐hour post‐transduction transduced cells and untransduced control cells (*n* > 15 wells) were insulted with 6‐mM NaIO_3_ and 12‐h post‐insult cells underwent a mitochondrial stress test. (P) Basal and (Q) maximal oxygen consumption rates (OCRs), (Q) spare respiratory capacity (SRC) and (R) ATP production are indicated. (S) OCR rescue by AAV2/2‐ophNdi1, post‐rotenone treatment. OCR was normalised to protein. Mitochondrial stress tests on pRPE1–3 were performed on separate occasions ^*^ *p* < .05; ^**^ *p* < .01; ^***^ *p* < .001; paired *t*‐test

**FIGURE 4:**
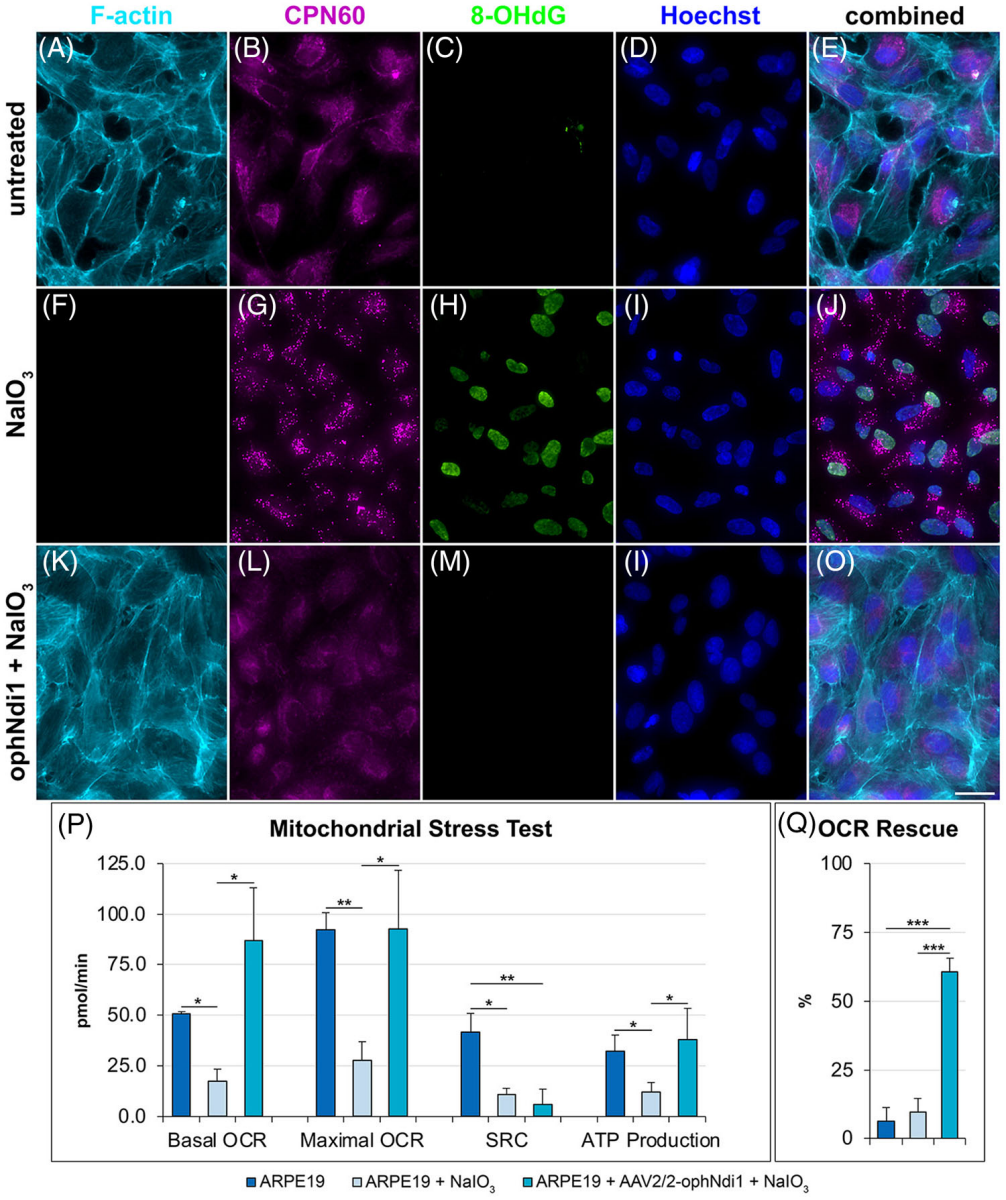
Rescue of ARPE19 cells insulted with NaIO_3_. 5.0 × 10^4^ ARPE19 cells were transduced with AAV2/8‐ophNdi1 5‐h post‐seeding; MOI = 5.4 × 10^5^ (K–O). Twenty‐eight‐hour post‐transduction cells were insulted with 5‐mM NaIO_3_ (F–O) and 24‐h post‐insult cells were fixed and stained with Phalloidin‐iFluor 647 (F‐actin, light blue), and CPN60 (mitochondrial marker, magenta) and 8‐OHdG‐Alexa Fluor 488 (oxidative stress marker, green) immunocytochemistries; nuclei were counterstained with Hoechst (nuclear stain, dark blue). AAV2/8‐ophNdi‐treated and NaIO_3_‐insulted cells (K–O) were compared to untreated (A–E) and untreated and NaIO_3_‐insulted cells (F–J). Expression of ophNdi1 provides clear rescue as the insulted cells treated with the virus have a similar phenotype to untreated control cells. Scale bar (O): 25 μm. (P and Q) 5.0 × 10^4^ ARPE19 cells were seeded into XFe96 Seahorse plates (*n* = 3). The following day a minimum of five wells were transduced with AAV2/2‐ophNdi1 (MOI = 3.4 × 10^5^). Twenty‐eight‐hour post‐transduction transduced cells and a minimum of 16 wells of untransduced cells were insulted with 5‐mM NaIO_3_ and 12 h post‐insult cells underwent a mitochondrial stress test using an XFe96 Seahorse. (P) Basal and maximal oxygen consumption rates (OCRs), spare respiratory capacity (SRC) and ATP production are indicated. (Q) Rescue of OCR by AAV2/2‐ophNdi1 post‐rotenone treatment is also indicated. OCRs are normalised to protein. NaIO_3_ insult reduced basal OCRs, maximal OCRs, SRC and ATP production significantly compared to cells that received no insult (by 66.0%, *p* < .05; 69.8%, *p* < .01; 74.4%, *p* < .001 and 62.1%, *p* < .05, respectively). Notably, SRC (the difference in maximal and basal OCR) is further reduced by AAV2/2‐ophNdi1 as the increase in basal OCR caused by the ophNdi1 expression is greater than the increase in maximal OCR. OCR was normalised to protein. ^*^ *p* < .05; ^**^ *p* < .01; ^***^ *p* < .001; 2‐sample *t*‐test

We tested NDI1 and ophNdi1, which target mitochondrial dysfunction, known to be a key factor in dry AMD. Robust benefit was demonstrated with multiple AAV‐delivered NDI1/ophNdi1 vectors and doses in the *Cfh^−/−^* and NaIO_3_‐induced mouse models as well as two cell models. The study represents the first demonstration globally of functional benefit in vivo in dry AMD models provided by a gene therapy directly targeting mitochondrial function.

## CONFLICT OF INTEREST

SMW, NC, MC, PFK and GJF are inventors on patent no. 10220102.

## Supporting information

Supporting Material

Supporting Material

## References

[1] Wong WL , Su X , Li X , et al. Global prevalence of age‐related macular degeneration and disease burden projection for 2020 and 2040: a systematic review and meta‐analysis. Lancet Global Health. 2014;2:e106–e116. 25104651 10.1016/S2214-109X(13)70145-1

[2] Cabral de Guimaraes TA , Daich Varela M , Georgiou M , Michaelides M . Treatments for dry age‐related macular degeneration: therapeutic avenues, clinical trials and future directions. Br J Ophthalmol. 2022;106:297–304. 33741584 10.1136/bjophthalmol-2020-318452 PMC8867261

[3] Kaarniranta K , Uusitalo H , Blasiak J , et al. Mechanisms of mitochondrial dysfunction and their impact on age‐related macular degeneration. Prog Retin Eye Res. 2020;79:100858. 32298788 10.1016/j.preteyeres.2020.100858 PMC7650008

[4] Ferrington DA , Fisher CR , Kowluru RA . Mitochondrial defects drive degenerative retinal diseases. Trends Mol Med. 2020;26:105–118. 31771932 10.1016/j.molmed.2019.10.008 PMC6938541

[5] Coffey PJ , Gias C , McDermott CJ , et al. Complement factor H deficiency in aged mice causes retinal abnormalities and visual dysfunction. Proc Natl Acad Sci USA. 2007;104:16651–16656. 17921253 10.1073/pnas.0705079104 PMC2034255

[6] Toomey CB , Kelly U , Saban DR , Bowes Rickman C . Regulation of age‐related macular degeneration‐like pathology by complement factor H. Proc Natl Acad Sci USA. 2015;112:e3040–e3049. 25991857 10.1073/pnas.1424391112 PMC4466717

[7] Yagi T , Seo BB , Nakamaru‐Ogiso E , et al. Possibility of transkingdom gene therapy for complex I diseases. Biochim Biophys Acta. 2006;1757:708–714. 16581014 10.1016/j.bbabio.2006.01.011

[8] Talla V , Koilkonda R , Guy J . Gene therapy with single‐subunit yeast NADH‐ubiquinone oxidoreductase (NDI1) improves the visual function in experimental autoimmune encephalomyelitis (EAE) mice model of multiple sclerosis (MS). Mol Neurobiol. 2020;57:1952–1965. 31900864 10.1007/s12035-019-01857-6

[9] Sorsby A . The nature of experimental degeneration of the retina. Br J Ophthalmol. 1941;25:62–65. 18169747 10.1136/bjo.25.2.62 PMC1143263

[10] Mizota A , Adachi‐Usami E . Functional recovery of retina after sodium iodate injection in mice. Vision Res. 1997;37:1859–1865. 9274771 10.1016/s0042-6989(97)00015-1

