# Supplementary material for: AAV‐mediated gene therapy improving mitochondrial function provides benefit in age‐related macular degeneration models
Source: Clin Transl Med. 2022 Aug 21;12(8):e952. doi: 10.1002/ctm2.952 (PMC9393074; doi:10.1002/ctm2.952)
Supplement: Supplementary file 1 — Supporting Material [file CTM2-12-e952-s002.docx]

Supplemental Figures and Tables

|  | 6 month C57BL/6J | 6 month *Cfh^-/-^* | Reduction in *Cfh^-/-^* |
| --- | --- | --- | --- |
| Rod b (μV) | 407±138 (n=24) | 317±83.0 (n=14) | 22% |
| Max b (μV) | 672±259 (n=24) | 496±123 (n=14) | 26% |
| SFC b (μV) | 114±50.8 (n=25) | 67.0±21.3 (n=22) | 41% |
|  | 9 month C57BL/6J | 9 month *Cfh^-/-^* | Reduction in *Cfh^-/-^* |
| Rod b (μV) | 288±112 (n=19) | 210±81.8 (n=33) | 27% |
| Max b (μV) | 472±219 (n=19) | 329±112 (n=33) | 30% |
| SFC b (μV) | 71.4±32.7 (n=18) | 41.6±12.5 (n=33) | 42% |
|  | 12 month C57BL/6J | 12 month *Cfh^-/-^* | Reduction in *Cfh^-/-^* |
| Rod b (μV) | 275±88.5 (n=27) | 217±61.4 (n=14) | 21% |
| Max b (μV) | 447±121 (n=25) | 325±96.3 (n=14) | 27% |
| SFC b (μV) | 74.9±33.5 (n=25) | 53.5±17.8 (n=14) | 29% |
|  | 18 month C57BL/6J | 18 month *Cfh^-/-^* | Reduction in *Cfh^-/-^* |
| Rod b (μV) | 302±107 (n=20) | 211±50.7 (n=10) | 30% |
| Max b (μV) | 509±226 (n=20) | 328±95.7 (n=10) | 36% |
| SFC b (μV) | 96.8±55.3 (n=20) | 53.2 (n=10) | 45% |

TABLE S1. ERG analysis of C57BL/J6 and *Cfh^-/-^* mice. Rod b, Max b and single flash cone (SFC) b ERG responses and percentage reduction in ERGs observed in *Cfh^-/-^* mice compared to C57BL/6J mice at 6 months, 9 months and 18 months of age. n numbers are indicated.

FIGURE S1. Relative mRNA expression levels from AAV2/8-Ndi1, AAV2/5-ophNdi1 and AAV2/8-ophNdi1 in murine retina. Adult *Cfh^-/-^* mice were subretinally injected with 3x10^9^ vg of each virus. Expression of NDI1 and ophNdi1 from the AAV vectors was compared in retinal RNA samples four weeks post-injection. Relative expression levels were 100.0±70.13%, 305.4±143.1% and 263.7±247.5% respectively, indicating that expression levels from optimised AAV2/5-ophNdi1 and AAV2/8-ophNdi1 are 3.0-fold and 2.6-fold higher than from AAV2/8-Ndi1. ** p<0.01; 2-sample *t*-test


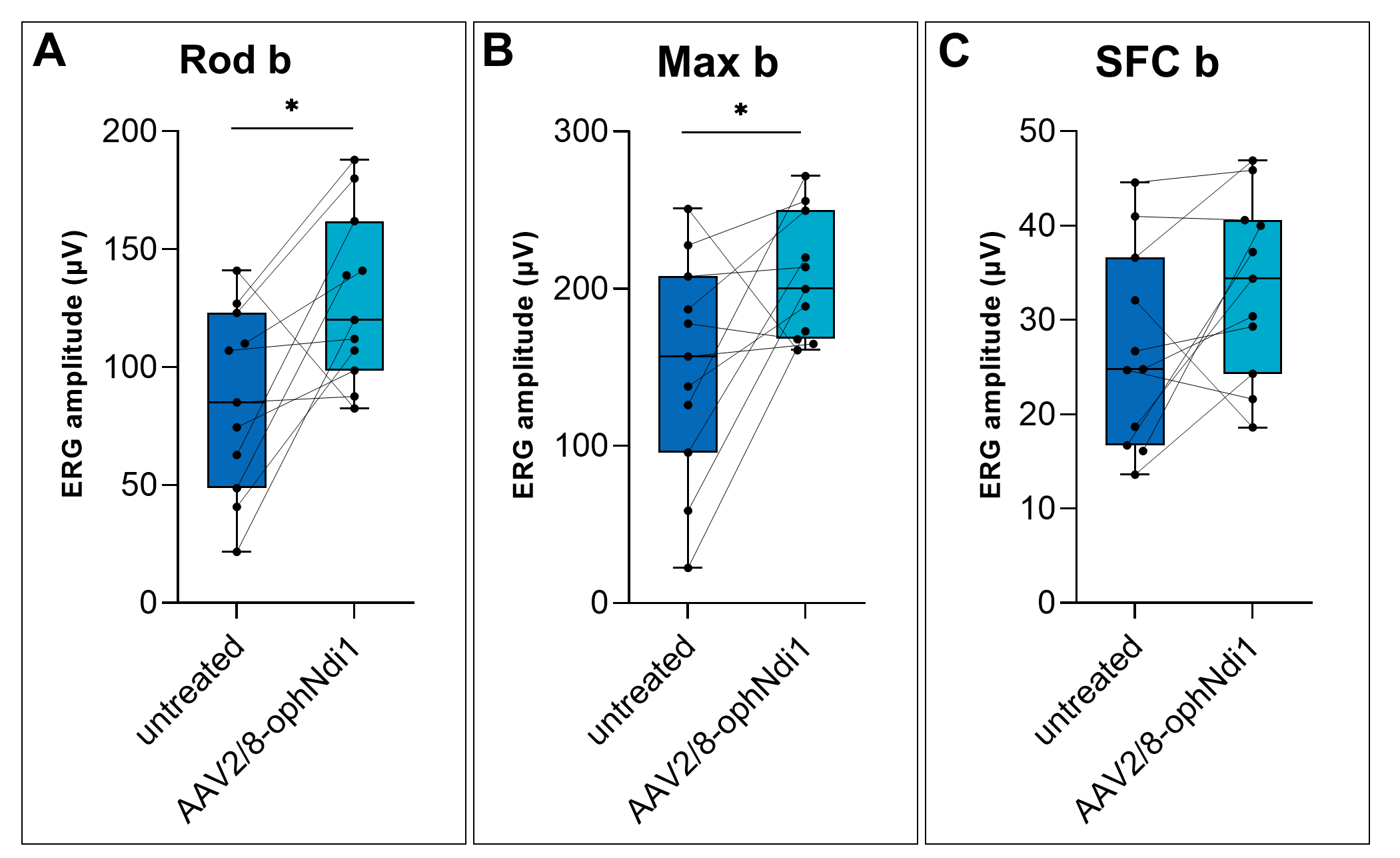


FIGURE S2. Rescue of *Cfh^-/-^* mice with AAV2/8-ophNdi1. Two-month *Cfh^-/-^* mice were subretinally injected with 7.5 x 10^7^ vg AAV2/8-ophNdi1 in one eye, while the contralateral eye was injected with 7.5 x 10^7^ empty capsids (n=11). ERGs of treated eyes compared to untreated contralateral eyes at 9 months showed significantly greater Rod b (A, 85.58±39.17 μV vs 128.9±36.15 μV), Max b (B, 150.0±70.82 μV vs 206.2±39.52 μV) and SFC b (C, 26.87±10.52 μV vs 33.56±9.571 μV) responses. * p<0.05; paired *t*-test.


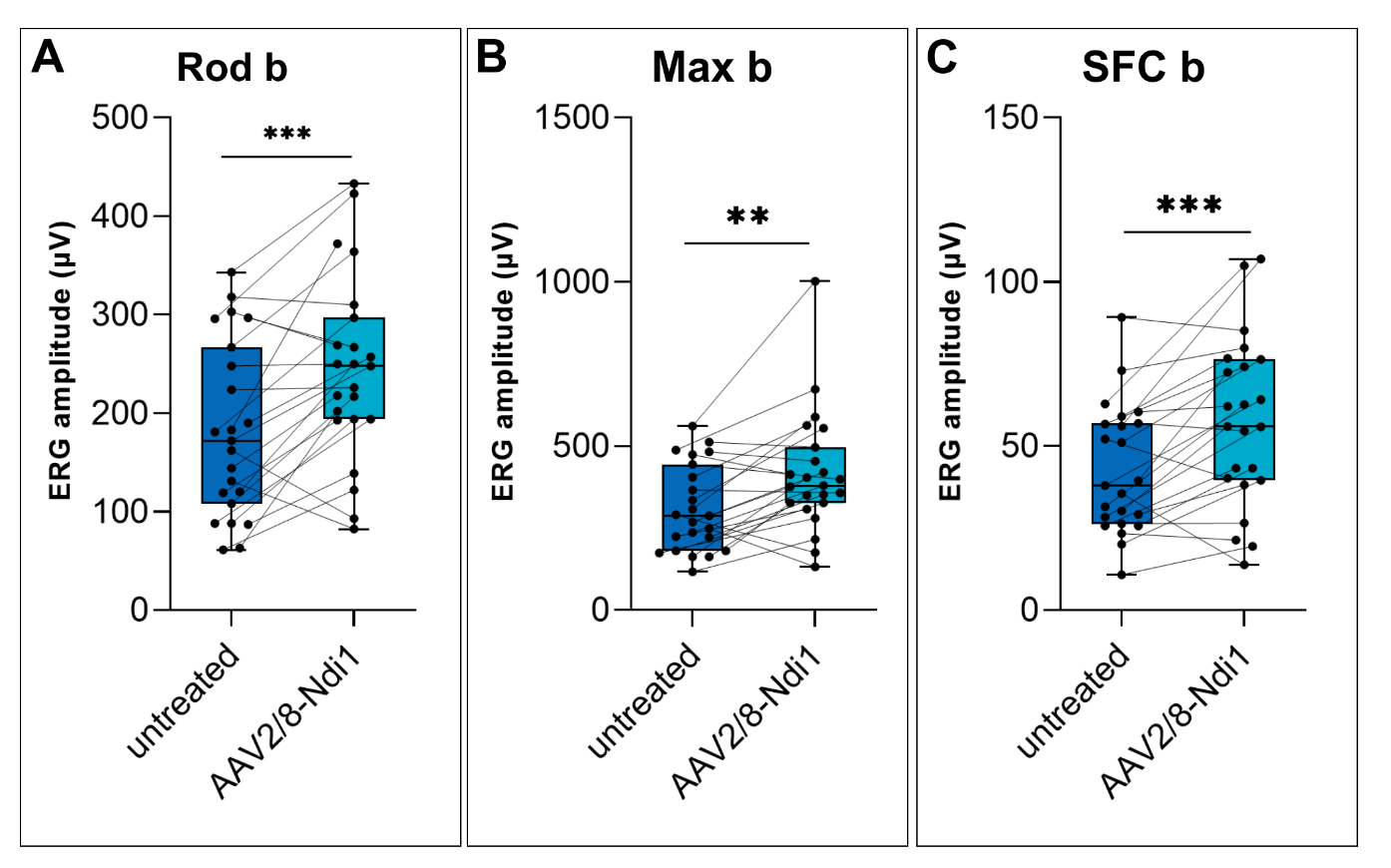


FIGURE S3. Rescue of *Cfh^-/-^* mice with AAV2/8-Ndi1. Two-month *Cfh^-/-^* mice were subretinally injected with 7.5 x 10^8^ vg AAV2/8-Ndi1 in one eye, while the contralateral eye remained uninjected (n=23). ERGs of treated eyes compared to untreated contralateral eyes at 9 months showed significantly greater Rod b (A, 182.3±88.43 μV vs 244.4±93.7 μV), Max b (B, 310.0±131.7 μV vs 415.0±181.9 μV) and SFC b (C, 42.7±19.4 μV vs 57.3±25.5 μV) responses. ** p<0.01; *** p<0.001; paired *t*-test.


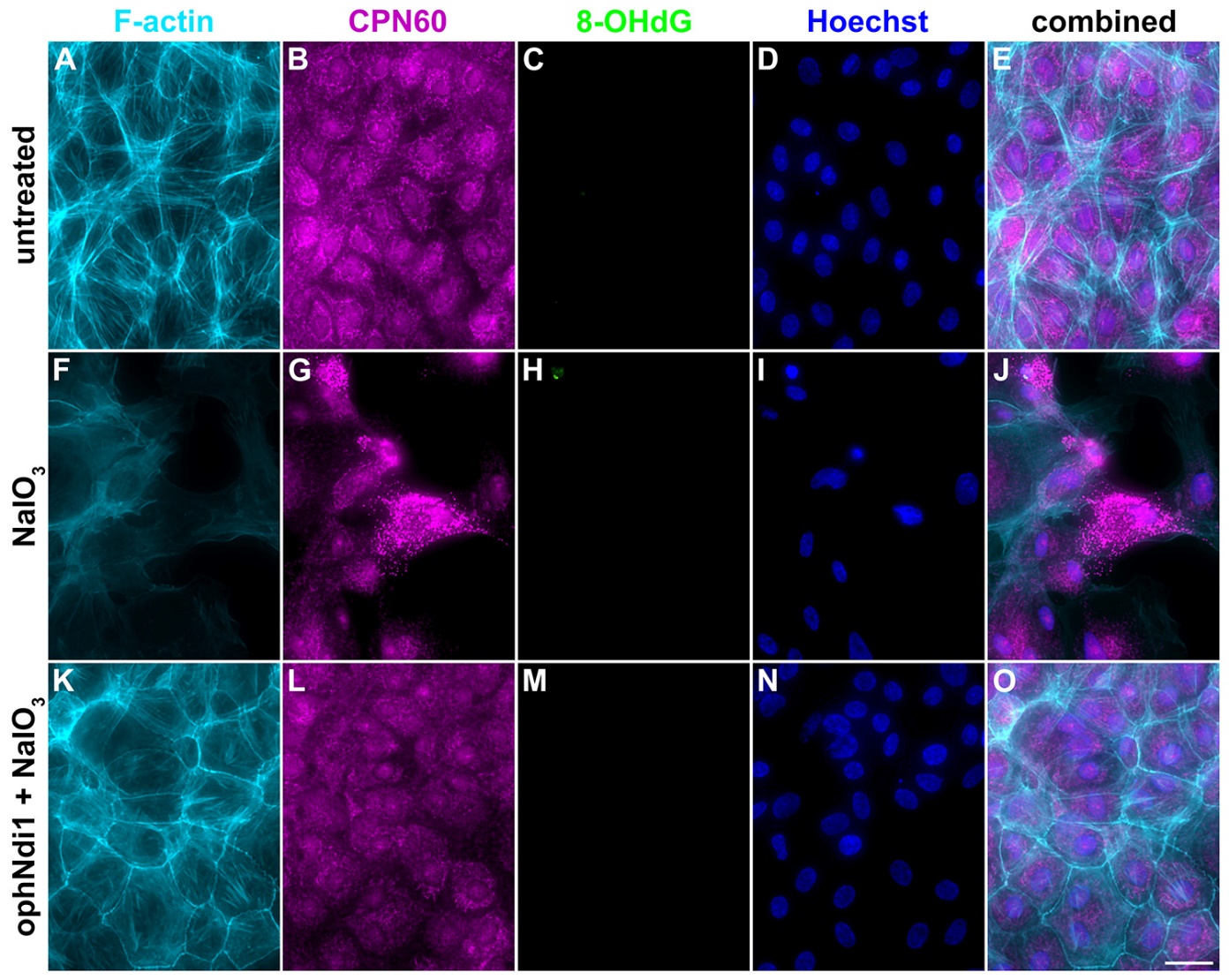


FIGURE S4. Rescue of primary porcine RPE (pRPE) cells insulted with NaIO_3_ at 48 hr post-insult. 7.5 x 10^4^ primary porcine RPE cells were transduced with AAV2/8-ophNdi1 5 hr post-seeding; MOI=5.4 x 10^5^ (K-O). 28 hr post-transduction cells were insulted with 5 mM NaIO_3_ (F-O) and 48 hr post-insult cells were fixed and stained with Phalloidin-iFluor 647 (F-actin, light blue), and CPN60 (mitochondrial marker, magenta) and 8-OHdG-Alexa Flour 488 (oxidative stress marker, green) immunocytochemistries; nuclei were counterstained with Hoechst (nuclear stain, dark blue). AAV2/8-ophNdi-treated and NaIO_3_-insulted cells (K-O) were compared to untreated (A-E) and untreated and NaIO_3_-insulted cells (F-J). Significant cells death can be observed in insulted cells that have not been treated with AAV2/8-ophNdi. However, as per Fig. 5 which showed rescue 24 hr post-insult, expression of ophNdi1 provides clear rescue from NaIO_3_ insult and insulted cells treated with the virus have a similar phenotype to untreated control cells. Scale bar (O): 25 μm.


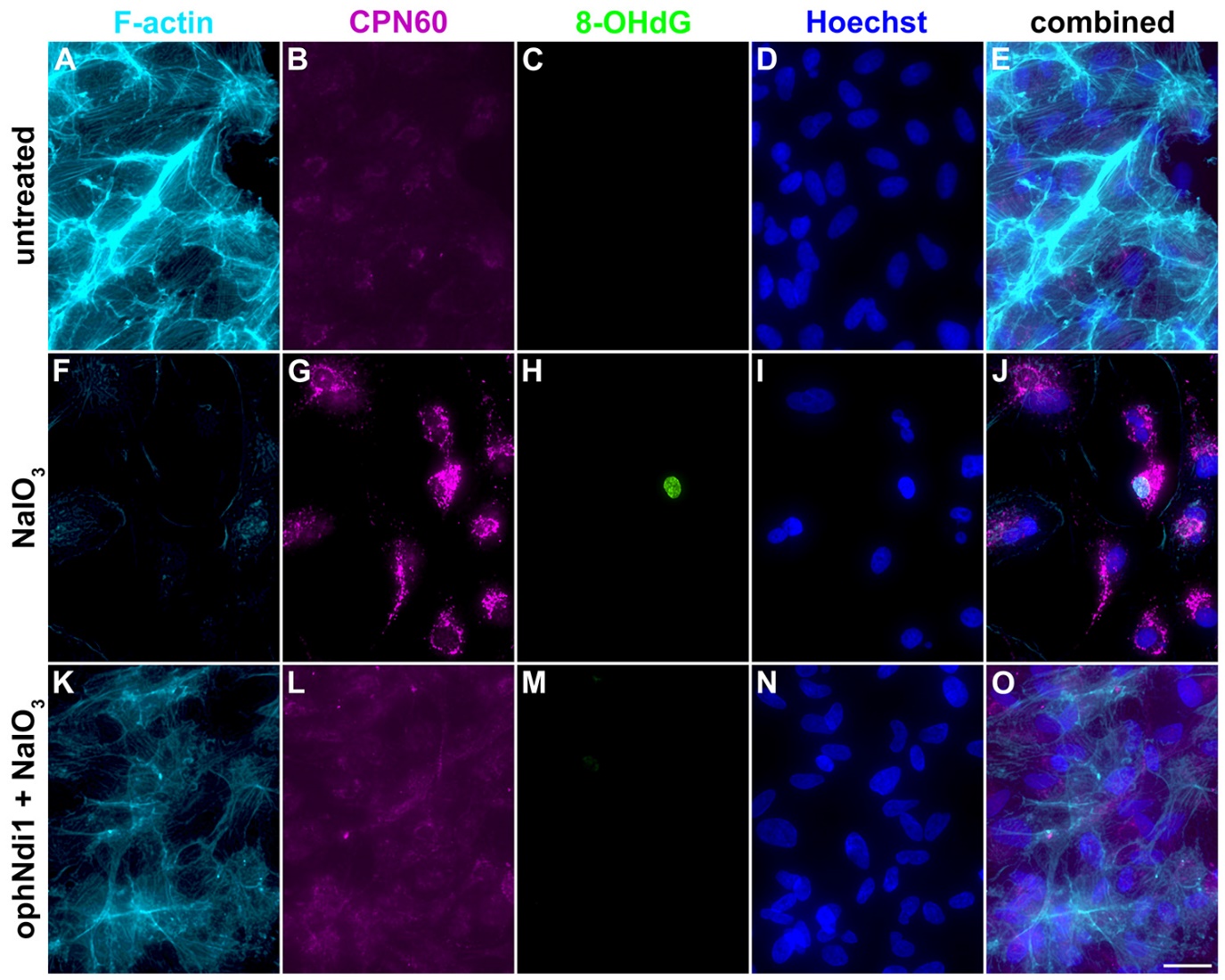


FIGURE S5. Rescue of ARPE19 cells insulted with NaIO_3_ at 48 hr post-insult. 5.0 x 10^4^ ARPE19 cells were transduced with AAV2/8-ophNdi1 5 hr post-seeding; MOI=5.4 x 10^5^ (K-O). 28 hr post-transduction cells were insulted with 5 mM NaIO_3_ (F-O) and 48 hr post-insult cells were fixed and stained with Phalloidin-iFluor 647 (F-actin, light blue), and CPN60 (mitochondrial marker, magenta) and 8-OHdG-Alexa Flour 488 (oxidative stress marker, green) immunocytochemistries; nuclei were counterstained with Hoechst (nuclear stain, dark blue). AAV2/8-ophNdi-treated and NaIO_3_-insulted cells (K-O) were compared to untreated (A-E) and untreated and NaIO_3_-insulted cells (F-J). Significant cells death can be observed in insulted cells that have not been treated with AAV2/8-ophNdi. However, as per Fig. 5 expression of ophNdi1 provides clear rescue and insulted cells treated with the virus have a similar phenotype to untreated control cells. Scale bar (O): 25 μm.

| pmol/min | basal OCR | max OCR | SRC | ATP |
| --- | --- | --- | --- | --- |
| ARPE19 | 50.4±0.582 | 92.0±8.54 | 41.6±9.5 | 32.2±15.3 |
| ARPE19 + NaIO_3_ | 17.1±6.12 | 27.8±9.24 | 10.7±3.14 | 12.2±4.70 |
| ARPE19 + ophNdi + NaIO_3_ | 86.8±26.0 | 92.8±29.0 | 5.94±7.34 | 38.0±15.3 |

TABLE S2. Rescue of mitochondrial function in NaIO_3_-induced ARPE19 model. 5.0 x 10^4^ ARPE19 cells were seeded into XFe96 Seahorse plates (n=3).The following day a minimum of 5 wells were transduced with AAV2/2-ophNdi1 (MOI=3.4 x 10^5^). 28 hr post-transduction transduced cells and a minimum of 16 wells of untransduced cells were insulted with 5 mM NaIO_3_ and 12 hr post-insult cells underwent a mitochondrial stress test using the XFe96 Seahorse. Basal and maximal (max) oxygen consumption rates (OCRs), spare respiratory capacity (SRC) and ATP production are indicated in ARPE19 (control cells), ARPE19 cells insulted with NaIO_3_ and ARPE19 cells treated with AAV2/2-ophNdi1 and insulted with NaIO_3_. OCRs are normalised to protein.

**METHODS AND MATERIALS**

**1 Study design**

Mitochondrial complex 1 equivalent NDI1 gene and an optimised NDI1 gene, ophNdi1 were delivered via recombinant AAV to models of dry AMD: the *Cfh^-/-^* mouse, NaIO_3_-induced mouse and primary pRPE and ARPE19 cells insulted with NaIO_3_. Functional effects were determined using physiological readouts, ERG and OKR, histological analysis and cellular assays for ROS and NADH oxidation. *In vitro* assays included mitochondrial function, ROS and morphological readouts.

**2 Plasmid constructs, AAV production and *in vivo* analysis of relative expression levels**

Plasmid constructs used to generate recombinant AAVs, AAV-Ndi1 and AAV-ophNdi1 are described (patent no 10220102; Chadderton *et al*., *Eur J Hum Genet.* 2013). AAV2/5-ophNdi1, AAV2/8-Ndi1 and AAV2/8-ophNdi1 and AAV2/2-ophNdi1, were generated by helper virus-free, triple transfection as described (O’Reilly *et al*., *Am J Hum Genet.* 2007). Notably, AAV2/2 serotype was utilised in cellular models as this serotype transduces ARPE19 and porcine RPE cells efficiently. However, AAV2/5 and AAV2/8 were used in murine models as these serotypes are known to transduce both RPE and photoreceptor cells; the cells affected in AMD (Trapani et al., EMBO Mol Med. 2014; Palfi et al., Mol Ther Methods Clin Dev 2015). The general CAG and CMV promoters were utilised to drive ophNdi1 expression, as these express highly in the target cell types (Fischer et al., Mol Ther. 2017; Palfi et al., Mol Ther Methods Clin Dev 2015). Genomic titres (vg/ml) were determined by RT-qPCR. 3.0 x 10^9^ vg of AAV2/8-Ndi1 or AAV2/5-ophNdi1 or AAV2/8-ophNdi1 were injected subretinally into six-week *Cfh^-/-^* mice (n=4-5 eyes per group). Four weeks post-injection retinas were snap frozen and total RNA isolated (Qiagen; Millington-Ward *et al*., *Mol Ther*. 2011; Chadderton *et al*., *Mol Ther*. 2009; Palfi *et al*., *Mol Ther Methods Clin Dev*. 2015). RNA was also isolated from age-matched uninjected control *Cfh^-/-^* retinas (n=3). RT-qPCR was undertaken using plasmid DNA containing either NDI1 or ophNdi1 for standard curves of copy number and results normalised using β-actin (*Actb*).

**3 pRPE and ARPE19 NaIO_3_–induced model**

Primary pRPE cells were isolated and maintained from mature pig eyes (n=3 pigs; Arnault *et al*., *Plos One*. 2013). ARPE19 cells, obtained from the ATCC (CRL-2302), were maintained as described. 5.0 x 10^4^ cells were seeded into XFe96 Seahorse plates (n=3; Agilent). Five wells were transduced with AAV2/2-ophNdi1 (MOI=3.4 x 10^5^) 24 hr later. 28 hr post-transduction, transduced and untransduced cells (>15 wells per group) were insulted with 6 mM NaIO_3_ (pRPE cells) or 5 mM NaIO_3_ (ARPE19 cells) and 12 hr post-insult cells underwent a mitochondrial stress test using an XFe96 Seahorse (Agilent). Injection cycles were 5x for basal OCR, 5x following oligomycin (1.0 μM), 5x following FCCP (1.0 μM), 5x following rotenone (0.5 μM) and 5x following antimycin A (0.5 μM) injections (Maloney *et* *al.*, *Front Neurosci*. 2020; Finnegan *et al*., *In J Mol Sci*. 2022). Basal and maximal OCRs, ATP production and SRC were determined as well as OCR rescue post-rotenone treatment (measurement 20-25/measurement 15-25*100%). Values were normalised to protein by Bradford assay (Thermo Scientific). Absorbance was determined on a FLUOstar OPTIMA at 595 nm (BMG Labtech). 7.5 x 10^4^ pRPE cells or 5.0 x 10^4^ ARPE19 cells were seeded onto 8-well imaging slides (Miltenyi Biotec). 5 hr post-seeding cells were transduced with AAV2/8-ophNdi1 (MOI=5.4 x 10^5^). 28 hr post-transduction cells were insulted with 5 mM NaIO_3_ and 24 hr or 48 hr post-insult cells were fixed in 4% paraformaldehyde in PBS at RT for 20 min. Cells were stained and analysed (see below).

**4 Subretinal injections and Electroretinography**

C57BL/6J, *Cfh^-/-^* on a pure C57BL/6J background and 129 S2/SvHsd mice (Harlan UK Ltd) were maintained under specific pathogen-free conditions. Animal experiments were performed in strict compliance with the European Union Regulations 2012 (S.I. no. 543 of 2021) and the Association for Research in Vision and Ophthalmology (ARVO) statement for use of animals. Subretinal injections, in 3 μl volumes, were performed on two-month mice (O’Reilly *et al*., *Am J* *Hum Genet*. 2007). 7.5 x 10^8^ vg of AAV2/8-Ndi1 was injected *Cfh^-/-^* mice (n=23), while contralateral eyes remained uninjected or received 7.5 x 10^8^ empty capsids. 5.7 x 10^8^ vg of AAV2/5-ophNdi1 was injected (n=14), while fellow eyes remained uninjected. 1.0 x 10^7^ vg of AAV2/8-ophNdi1 was injected (n=12), while fellow eyes received 1.0 x 10^7^ empty viral capsids–retinas from n=5 of these and n=2 C57BL/6J mice were analysed histologically at one year of age (below). 7.5 x 10^7^ vg of AAV-ophNdi1 was injected (n=11), while fellow eyes received 1.0 x 10^7^ empty viral capsids. ERG analysis was performed as described O’Reilly *et al*., *Am J* *Hum Genet*. 2007. ERG responses from treated eyes were compared to fellow eyes (paired *t*-tests).

**5 ROS levels in retinal samples**

Two-month *Cfh^-/-^* mice were injected with 2.4 x 10^9^ vg AAV2/8-Ndi1 (n=10). Three months post-injection, mice were sacrificed, and retinal cells dissociated (Chadderton *et al*., *Mol Ther*. 2009). Retinas (n=18) from age-matched control *Cfh^-/-^* mice were also dissociated. Cell pellets were suspended in 250 μl HBSS with 2.5 μl DNase (Sigma D5025) and 2 μl CellRox^TM^ Green Reagent (Thermo Fisher Scientific). Retinal cells were incubated in the dark at 37ᵒC for 2.5 hr. 0.5 μl DRAQ5^TM^ (BD Biosciences, San Diego, CA) was added to aid in gating live cells on a flow cytometer (BD Accuri^TM^ C6, BD Biosciences). Median levels of CellRox^TM^, representing relative ROS levels, were recorded. 2-sample *t*-tests were performed to compare means.

**6 NADH oxidation in retinal samples**

NADH oxidation activities in retinas from 14-month *Cfh^-/-^* mice, subretinally injected at 2 months with 7.5 x 10^7^ vg AAV2/8-ophNdi1 in one eye and 7.5 x 10^7^ empty capsids in the contralateral eye (n=4), were compared to each other and to NADH oxidation activities in retinas from age-matched C57BL/6J mice. Retinas were snap frozen and mitochondria isolated (Chadderton *et al*., *Eur J Hum Genet*. 2013). NADH oxidation activities on mitochondria (Spinazzi *et al*., *Nat Protoc*. 2012) were measured twice per sample and duplicate readings averaged. Means were compared with paired *t*-tests.

**7 NaIO_3_–induced murine model**

Two-month 129 S2/SvHsd mice were subretinally injected in one eye with 7.5 x 10^7^ vg AAV2/8-ophNdi1 and 1.0 x 10^8^ vg AAV2/2-CAG-EGFP,^75^ and in the other eye with 1.0 x 10^8^ AAV2/2-CAG-EGFP (n=8). At five months mice were injected via tail-vein with 22mg/kg NaIO_3_ in 0.9% NaCl_2_. Mice underwent ERG analysis at 7 days and OKR analysis one month post-insult.^63^ OKR spatial frequency thresholds were measured blind on three occasions using a virtual optokinetic system (OptoMotry, CerebralMechanics, Lethbridge). Treated and untreated eyes were compared using paired *t*-tests. Additionally, three months post-subretinal injection of 5.7 x 10^8^ vg AAV2/5-ophNdi1 plus 1.0 x 10^8^ vg AAV2/2-CAG-EGFP and 1.0 x 10^8^ AAV2/2-CAG-EGFP in the contralateral eye (n=6), mice received 50mg/kg NaIO_3_ in 0.9% NaCl_2_ via tail-vein. Retinas were processed for histology 7 days post-insult.

**8 Immunohistochemistry and microscopy**

Mice were sacrificed, eyes enucleated and fixed in 4% paraformaldehyde in PBS at 4°C overnight, washed in PBS, cryoprotected and cryosectioned (12 μm). Retinal sections adjacent to the optic nerve head (±200 μm) were used. Immunohistochemistry and immunocytochemistry (O’Reilly *et al*., *Am J Hum Genet*. 2007; Millington-Ward *et al*., *Mol Ther*. 2011; Chadderton *et al*., *Mol Ther*. 2009; Palfi *et al*., *Mol Ther Methods Clin Dev*. 2015) utilised Phalloidin-iFluor 647 (1/1000 dilution, ab176759, Abcam), anti-8-OHdG-Alexa Fluor 488 (1/200 dilution, sc-393871, SantaCruz), anti-CPN60 (1/2000 dilution, RPCA-HSP60, EnCor Biotechnology Inc.) and anti-ARR3 (1/200 dilution, AB15282, Merck) primary antibodies. Secondary antibodies conjugated with Cy3 (Jackson ImmunoResearch Europe Ltd.) were used at 1/400 dilution. Nuclei were counterstained with DAPI (retinas) or Hoechst (cells). Fluorescent microscopy was undertaken utilising an Olympus IX83 (Mason Technology, Dublin; Millington-Ward *et al*., *Sci Rep*. 2020). Cone cells were manually marked on an overlaid layer in Adobe Photoshop before quantifying in ImageJ (NIH; Maryland) with sections from two slides analysed per retina.

**9 Transmission electron microscopy (TEM)**

Eyes from 1-year old *Cfh^-/-^* mice (n=8) and C57BL/6J mice (n=4) were fixed overnight in 2.5% glutaraldehyde and 0.1M PIPES pH7.0. The following day eyes were placed in PBS. Tissue blocks of central retina were post-fixed in 1% osmium tetroxide, dehydrated through graded concentrations of ethanol and embedded in modified Spurr’s resin (Polysciences).

**10 Statistical analysis**

Statistical analysis was performed using GraphPad 9.3. *t*-tests were considered significant at p<0.05.
